# Multitargeted Effects of Vitexin and Isovitexin on Diabetes Mellitus and Its Complications

**DOI:** 10.1155/2021/6641128

**Published:** 2021-04-10

**Authors:** Ibrahim Luru Abdulai, Samuel Kojo Kwofie, Winfred Seth Gbewonyo, Daniel Boison, Joshua Buer Puplampu, Michael Buenor Adinortey

**Affiliations:** ^1^West African Centre for Cell Biology of Infectious Pathogens, College of Basic and Applied Sciences, University of Ghana, P.O. Box LG 54, Legon, Accra, Ghana; ^2^Department of Biomedical Engineering, School of Engineering Sciences, College of Basic and Applied Sciences, University of Ghana, P.O. Box LG77, Legon, Accra, Ghana; ^3^Department of Biochemistry, Cell and Molecular Biology, School of Biological Sciences, University of Ghana, Legon, Accra, Ghana; ^4^Department of Biochemistry, School of Biological Sciences, University of Cape Coast, Cape Coast, Ghana

## Abstract

**Background:**

Till date, there is no known antidote to cure diabetes mellitus despite the discovery and development of diverse pharmacotherapeutic agents many years ago. Technological advancement in natural product chemistry has led to the isolation of analogs of vitexin and isovitexin found in diverse bioresources. These compounds have been extensively studied to explore their pharmacological relevance in diabetes mellitus. *Aim of the Study*. The present review was to compile results from in vitro and in vivo studies performed with vitexin and isovitexin derivatives relating to diabetes mellitus and its complications. A systematic online literature query was executed to collect all relevant articles published up to March 2020.

**Results:**

In this piece, we have collected data and presented it in a one-stop document to support the multitargeted mechanistic actions of vitexin and isovitexin in controlling diabetes mellitus and its complications.

**Conclusion:**

Data collected hint that vitexin and isovitexin work by targeting diverse pathophysiological and metabolic pathways and molecular drug points involved in the clinical manifestations of diabetes mellitus. This is expected to provide a deeper understanding of its actions and also serve as a catapult for clinical trials and application research.

## 1. Introduction

Diabetes mellitus is a disorder characterized by persistent hyperglycemia, resulting from partial or complete pancreas damage leading to the imbalance in carbohydrate, lipid, and protein homeostases. A hyperglycemic state is the effect of impaired insulin secretion, insulin resistance, or both [1]. Medicinal plants and their isolated compounds have received huge attention due to their reported use in the management of diabetes mellitus (DM). Though several discoveries have been made concerning pharmacotherapies used for managing diabetes mellitus, its management is still a major challenge for healthcare providers. Several attempts to grapple with the disease have been confronted by humungous financial expenses. In the U.K, for instance, about 10% of the primary care prescribing budget is spent on treating diabetes, with a daily average of £2.2 m spent by the National Health Service (NHS) on prescriptions only [2]. The global economic cost as at 2014 was an estimated US$612 billion. In urban Ghana, the economic burden of diabetes is high with catastrophic effects on households [3]. Studies conducted by [4] revealed that the overall total cost estimate for diabetes treatment by individuals in Ghana was USD 10,485.11 with a total mean cost of USD 46.81 on monthly basis.

**Figure 1:**
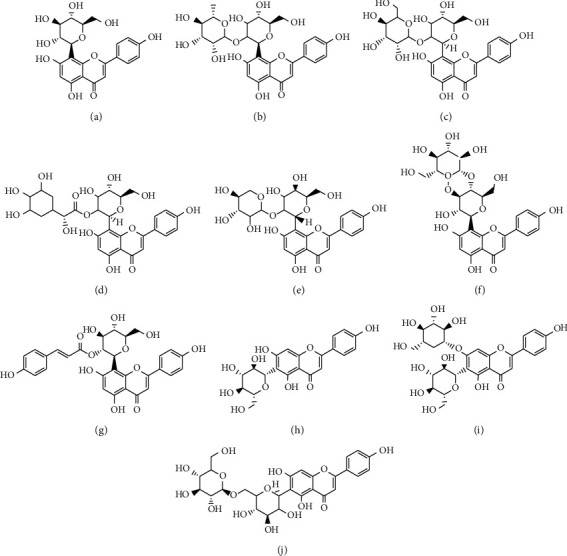
Structures of vitexin (apigenin-8-C-*β*-glucopyranoside) and its analogs. (a) Vitexin (apigenin-8-C-B-glucopyranoside). (b) Vitexin 2″-O-rhamnoside. (c) Vitexin 2″-O-glucoside. (d) 2″-O-galloylvitexin. (e) Vitexin 2-O-xyloside. (f) Vitexin 4″-O-glucoside. (g) Vitexin 2″-O-p-trans-coumarate. (h) Isovitexin (apigenin-6-C-B-glucopyranoside). (i) Isovitexin-7-O-glucoside.(j) Isovitexin 6″-O-glucoside.

Till date, there is no known antidote to cure diabetes mellitus despite the discovery and development of diverse pharmacotherapeutic agents many years ago. The high cost of these drugs and the unbearable side effect they come along with heighten the demand for natural antidiabetic products which may possess minimal to no side effects. Currently, consideration of two mono-*c*-glycosylflavones, vitexin and isovitexin, as drug candidates for diabetes mellitus and related complication [5], is a growing research interest amongst many scientists all over the world. Several reports are dotted on different publishing platforms, thus necessitating the need for a one-stop document that provides knowledge on past and recent advancement regarding studies on vitexin and its analogues (Figure 1) as drug candidates for diabetes mellitus. This review provides an appraisal of experimental data on vitexin and isovitexin in published works that could serve as the basis for research consideration in other preclinical studies and consequently clinical trials. In this piece, we methodically provide a comprehensive summary of the antidiabetic mechanisms involved in controlling persistent hyperglycemia to serve as evidence and also provide a deeper understanding of its actions. This is expected to act as a reference for further clinical applications and research.

## 2. Methodology

### 2.1. Search Strategy

A systematic online literature query was executed to collect all relevant articles published up to March 2020. The systematic search was conducted on selected databases such as ScienceDirect, PubMed, and Google Scholar with the singleword “vitexin” to obtain published studies to-date. Investigators were not contacted, and we did not attempt to identify unpublished data. The review was guided by Preferred Reporting Items for Systematic Reviews and Meta-Analyses (PRISMA) recommendations [6].

### 2.2. Articles Selection

**Figure 2:**
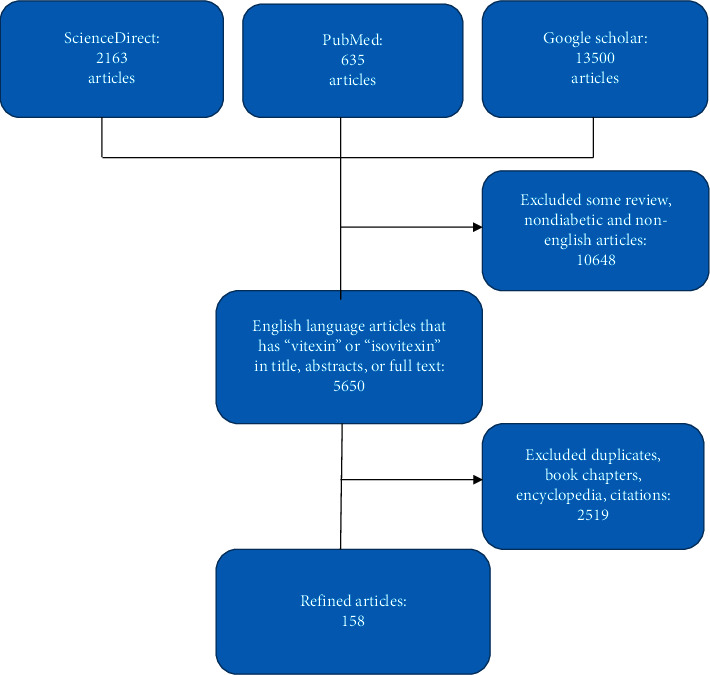
Flow chart of the search method and manuscripts selection.

The study articles were selected on the basis of an inclusion criteria stated as studies published in only English Language and those articles that have the defined keyword “vitexin” either in the title, abstract, whole text, or even the keywords. Articles on experimental screening with vitexin and isovitexin derivatives were of paramount importance. Review articles, abstracts, editorial/letters, conference, and proceedings were omitted in this systematic review. Also, articles unrelated to diabetes, except antioxidant, anti-inflammation, safety, and toxicological applications of vitexin were excluded. To further filter the articles, search results were screened manually and articles that comprise repeated data or studies duplicated by the search databases and some articles on phytochemical isolation of vitexin and isovitexin derivatives were excluded. The remaining articles represented the main source of information for the write-up.  Figure 2 is a flowchart showing the study selection process.

## 3. Results and Discussion

### 3.1. Natural Sources and Chemical Structural Properties of Vitexin and Isovitexin

Vitexin and isovitexin are mono-*C*-glycosylflavones found in diverse natural plant sources. Rapid technological advancement in natural product chemistry has led to the isolation of vitexin and its analogs. The isomer pair and their derivatives are ubiquitously available in natural sources such as insects [7], honey [8], fungus *Dichotomopilus funicola Y3* [9], and even plant tissues, including *Tetrastigma hemsleyanum* leaves [10], buckwheat sprouts [11], leaves of *Echinodorus scaber* [12], and others.

Vitexin (apigenin-8-C-*β*-glucopyranoside) and isovitexin (apigenin-6-C-*β*-glucopyranoside) analogs are mono-*C*-glycosylflavones with one sugar moiety attached to the flavone skeleton. In part of the C-glycoside structure of vitexin and isovitexin, the sugar moiety is connected right to the C6–C3–C6 aglycone skeleton through a C–C bond, which is reported to resist acid hydrolysis [13, 14]. It is confirmed that the sugar ring in a flavone-glucosyl moiety is attached through C-C bonds to the 8 position of the flavone nucleus and is approximately perpendicular to the flavone plane [15]. Vitexin and isovitexin appear as yellow crystalline chemical substances and are soluble in ethanol and dimethyl sulfoxide (DMSO) and mostly give yellow color solution when dissolved in an alkali medium [16]. Several studies have reported the biosynthesis of these class of compounds in the laboratory [17, 18].

### 3.2. Impact of Vitexin and Isovitexin on Drug Targets in Diabetes Mellitus and Its Complications

Vitexin and its analogs have been studied as an antihyperglycemic agent. Using rodents, oral administration of vitexin or isovitexin was reported to significantly decrease postprandial blood glucose content in a dose-dependent manner [19, 20]. Furthermore, isovitexin, in both normal and diabetic rats, demonstrated pronounced antihyperglycemic property and also attenuated a rise in blood glucose [21]. Hyperglycemia, caused mainly by insulin resistance and insensitive receptors, disturbs the metabolism of lipids and carbohydrates as well as proteins which can further progress to several micro and macrovascular complications. Examples of these complications include hypertension, dyslipidemia, neuropathy, nephropathy, and sexual impairments. Several targets are reported in literature to be useful points for antidiabetic drug candidates. These include pancreatic *β*-cell, lipid, and carbohydrate digesting enzymes, cell signaling molecules present in adipose tissue, angiotensin I-converting enzyme (ACE), free radicals, and glutamate transporters.

#### 3.2.1. Pancreatic *β*-Cell

It is now well noted that pancreatic *β*-cell functional deficit is a common feature of both type 1 and type 2 diabetes (t1d and t2d, respectively) [22]. In order to examine whether vitexin can protect pancreatic *β*-cell injury and apoptosis, a study on pancreatic *β*-cell malfunction was carried out. It was reported that vitexin treatment alleviates lipopolysaccharide- (LPS-) induced apoptosis and damage in islet tissue of rats and INS-1 cells via diminishing the levels of proinflammatory cytokines, TNF-*α*, and the high mobility group box 1 (HMGB1). Additional experiments proved that vitexin treatment blocked the initiation of P38 MAPK signaling pathways in LPS-mediated INS-1cells [23]. In NAFLD mice, vitexin also improved insulin signaling through upregulation of insulin receptor substrate-1 (IRS-1) and also its downstream target AKT [24]. In both in vivo and in vitro experiments, it has been demonstrated that vitexin improves pancreatic antioxidant enzyme (glutathionie reductase (GR) and superoxide dismutase (SOD) activities, decreases apoptosis and damage induced to islet tissue, stimulates islet cell regeneration, and significantly suppresses fasting blood glucose [23, 25]. SIRT6 has been mentioned as a key regulator of glucose homeostasis and serves as a therapeutic target for obesity and insulin resistant diabetes mellitus [26]. Singh et al. [27] identified vitexin as an active binder of the SIRT6 protein with binding affinity of 15.27. Later, vitexin acting as a SIRT6 modulator was confirmed using the affinity-based method [28]. Additionally, Rahim et al. [29] reported the antiobesity activities of isovitexins such as isovitexin, isovitexin-7-O-glucoside, and vitexin-2″-O-rhamnoside, which inhibited pancreatic lipase compared to vitexin which showed no activity. Macdonald [30] suggested that quinone reductase may participate in secretion of insulin. Following that, Boutin et al. [31] demonstrated the inhibition of quinone reductase 2 catalytic activity by flavonoids, such as vitexin-2″-O-rhamnoside, vitexin, and isovitexin. Available scientific experimental data have demonstrated that vitexin acts to protect against pancreatic *β*-cell injury and deceases apoptosis. It exercises this role by impacting positively on endogenous antioxidant status which mop out free radicals responsible for increased cell death.

#### 3.2.2. Alpha Amylase, Glucosidase, Aldose Reductase, Protein Tyrosine Phosphatase 1B, and Lipase

Enzymes are the main players in glucose metabolism and DM. Treatment of diabetes and its complications is achievable via obstructing key digestive enzymes that participate in lipid and starch digestion as well as metabolism [32]. There are reports on the use of vitexin against drug target enzymes in DM. In an experiment, isovitexin and vitexin revealed potency against *α*-amylase by reducing its activity, and vitexin binding to *α*-amylase showed stronger affinity (−7.54 kcal/mol) and stability than that of isovitexin (−5.61 kcal/mol) [33]. Several other studies suggest that vitexin, isovitexin-4′-methyl ether, isovitexin, and 2″-o-xylopyranosyl vitexin may serve as natural hypoglycemic and antidiabetic drugs due to their pronounced activities in *α*-glucosidase and blood glucose inhibition studies [19, 20, 34–39]. Additionally, acarbose and vitexin presented a synergistic inhibition on *α*-glucosidase, while vitexin alone uncompetitively repressed *α*-glucosidase activity and binding of vitexin brought about conformational changes in the enzyme structure [40]. Aldose reductase is one of the principal enzymes in the polyol metabolic pathway that catalyzes the reductive conversion of glucose to sorbitol, which is followed by fructose production catalyzed by the enzyme NADPH-dependent sorbitol dehydrogenase. Increased formation of fructose leads to increased dicarbonyl moiety which is a key player in AGE formation known to contribute to diabetic complications. It is also reported that excess intracellular sorbitol consequently causes osmotic damage to cells and leads to diabetic cataract complication [41]. Antidiabetic evaluation of C-glycosides revealed that vitexin and isovitexin displayed inhibitory activity against rat lens aldose reductase and human recombinant aldose reductase [42–44]. Protein tyrosine phosphatase 1B (PTP-1B) stimulates negative regulation of insulin and leptin signal transduction; thus, inhibition of this enzyme could serve as a target for diabetes mellitus control. A study by Choi and colleagues in 2013 demonstrated that vitexin and isovitexin possess antidiabetic potentials by its inhibiting effects on PTP-1B enzyme [42].

Generally, lipase enzymes catalyse the hydrolysis of ester bonds of triglycerides, and elevated serum lipase activity is implicated in t2ds [45]. Using the ligand fishing technique, isovitexin was identified as a lipase enzyme inhibitor [46]. Also, in lipase inhibitory assay, vitexin 2″-O-glucoside and vitexin 2″-O-rhamnoside exhibited antilipase activities at 131.48 *μ*M for vitexin 2″-O-glucoside and 114.49 *μ*M for vitexin 2″-O-rhamnoside [47]. Data enumerated allude to vitexin as a strong drug candidate for consideration in further drug discovery studies and consequently clinical trials for identifications of enzyme inhibitors.

#### 3.2.3. Adipose Tissue

Adipose tissue functions as the primary energy storage site and play additional role as an endocrine organ. Adipose tissue secretes adipokines (adiponectin, resistin, and leptin) which regulate energy homeostasis [48]. However, excessive adiposity is associated with adverse conditions such as obesity and DM [49]. In quest for the antidiabetic agent, vitexin was evaluated for its role in obstructing the pentose phosphate pathway as a novel therapeutic strategy for reducing the prevalence of obesity. Vitexin exhibited marked potency and reduced bodyweight, adipose accumulation and lipid storage in the liver, sugar consumption, and triglyceride production and regulated glycolysis by activation of the ERK 1/2 MAPK signaling pathway [50, 51].

One of the causes of obesity is unregulated genes, such as the CCAAT-enhancer-binding proteins (C/EBP-*α*), and peroxisome proliferator activated receptor *γ* (PPAR-*γ*) which stimulates lipid/glucose metabolism and crucial regulator of adipocyte differentiation. A study showed that vitexin had the highest activity and decreased C/EBP-*α* and PPAR-*γ* protein expression levels in 3T3-L1 cells [52]. Another study demonstrated that vitexin suppressed the phosphorylation of p38 and ERK involved in the discharge of inflammatory adipocytokines, although the expression of PPAR-*γ* was upregulated [53]. The report further mentioned that vitexin treatment increased phosphorylation of AMPK, TNF-*α*, and aP2 mRNA expression; whereas, the mRNA expression of C/EBP and SREBP1 was unaffected. Recently, Peng et al. [54] mentioned that vitexin significantly reduced lipid levels to normal, as well as reduced adipocyte size mediated by high-fat diet and consequently regulated lipid metabolism via AMPK-*α*, C/EBP-*α*, and fatty acid synthase in white adipose tissue and also significantly repressed fat buildup in adipocytes. In addition, vitexin was able to regulate intracellular lipogenesis and adipogenesis through anti-inflammatory mechanisms and the MEK/ERK pathway in the KK-Ay mouse model, and further in vitro studies with 3T3-L1 cells showed that vitexin inhibited inflammation-mediated lipogenesis with highly reduced levels of IL-6 and monocyte chemoattractant protein-1 (MCP-1) after vitexin treatment [55]. The evidences affirm the role of vitexin as an antiobesity drug agent.

#### 3.2.4. Angiotensin I-Converting Enzyme (ACE)

Hypertension extensively promotes the risk of both microvascular and macrovascular illnesses, including coronary artery disease and possibly neuropathy. Generally, inhibition of angiotensin I-converting enzyme (ACE) is currently considered to be a useful therapeutic approach in the treatment of high blood pressure. In an in vitro ACE-inhibitory activity experiment, isovitexin showed moderate activity (46%) and was more active than its 8-C-analog, vitexin (21%) [56]. In another study, the hypertensive action of vitexin was accompanied by an enhanced increase in rate and depth of respiration and a slight increase in mean arterial pressure of anaesthetized normotensive rats [57]. It was suggested that vitexin sustained hypotension due to its ganglion-inhibitory and anti-inflammatory, antihistaminic, antibradykinin, and antiserotonin effects. Vitexin is shown to have haemodynamic effects and caused decrease of mean aortic pressure, arterial and pulmonary capillary pressure, and heart rate [58]. According to Lu et al. [59], vitexin possesses the potential to protect against cardiac hypertrophy via Ca^2+^-induced calcineurin-NFATc3 and CaMKII pathways.

More recently, isovitexin vasodilatory effects were evaluated in an experiment by Tirloni and colleagues who reported that its potency was dependent on endothelium NO release, small conductance Ca^2+^-activated (SK KCa) potassium channels, and Kir6.1 ATP-sensitive K^+^ channels for vascular smooth muscle upregulation [60]. Also, vitexin strongly decreased blood pressure in volunteers and rabbits and increased urine flow, urinary sodium, and potassium excretions; hence, the hypertensive effect of vitexin was suggested to be most likely through its diuretic effects [61]. Ragone et al. [62] reported that the spasmolytic effect of *Aloysia citrodora* on rat duodenums is partly due to its vitexin, since vitexin noncompetitively inhibited the dose-response curves of acetylcholine and Ca^2+^ influx with higher affinity. Vitexin exhibits its spasmolytic properties via K^+^ channel activation and causes a concentration-dependent relaxation of the spontaneous contractions [63]. Experiments performed with purified isovitexin established that it successfully attenuated PDGF-mediated ERK1/2 activation and proliferation of rat aortic smooth muscle cells in culture, thus a novel candidate for atherosclerosis [64]. Furthermore, vitexin with antiarrhythmic potential significantly reduced isoproterenol-stimulated beating incidences at 3 and 10 mg/ml [65]. Mixing isovitexin with orientin produced a slightly significant vascular relaxation at high concentration of 60 mg/mL [66]. Data amassed signify the pharmacological role of vitexin and isovitexin in hypertension management which warrants further clinical research in that regard.

#### 3.2.5. Free Radicals and Glutamate Transporters (NMDAR, mGluR1, and mGlu5)

Diabetic neuropathy (DN) is one of the debilitating complications of DM. Several molecular and metabolic pathways responsible for diabetic neuropathy progression have been proposed for therapeutic investigations. The interruption in these pathways by vitexin and its derivatives redefine the medicinal application of these compounds in managing diabetic neuropathy. Vitexin and its derivatives mitigate DN via the following mechanisms: (1) antioxidant, (2) anti-inflammatory, and (3) gene or protein inhibitory activities. In PC12 cells, vitexin exhibited its neuroprotection via attenuation of cell apoptosis, neuroinflammation, oxidative stress, cytosolic calcium, and enhanced *β*-secretase 1 content [67, 68]. In addition, vitexin selectively modulated the expression of genes involved in the antioxidant pathway, glutamate transporters (NMDAR, mGluR1, and mGlu5), maintained cholesterol homeostasis and reduced ER stress to offer neuroprotection [69, 70]. Helms found that vitexin interacted with E2-2K, implicated in neurodegenerative diseases, at its E1 interacting site [71]. Also, according to Yang et al. [72], vitexin attenuates neuronal cell death through modification of Bcl-2 and Bax expressional balance, cleavage of poly (ADP-ribose) polymerase and caspase 3, and interferes with the calcium influx pathway in cultured cortical neurons. Further investigations showed that vitexin acts through PI3K/Akt, HIF-1*α*, VEGF, and p38 MAPK signaling paths, improves phosphorylation of PI3K and Akt, and concurrently blocks Bax/Bcl-2 ratio and caspase-3 activity in mice and SH-SY5Y cells [72]. Also, the use of vitexin prevented bradykinesia [73, 74]. Additionally, vitexin and isovitexin caused activation of Nrf2, enhanced the activity of HO-1, inhibited neutrophils activation and elevation of inflammatory cytokine (MAPK and NF-*κ*B) and ROS, and suppressed activated nucleotide-binding domain and NLRP3 inflammasome [75, 76]. In neuro-2a cells, vitexin blocks oxidative stress, facilitated damage by boosting the expression of the Nrf-2/HO-1 pathway, inducing glutamate transporter expression, downregulation of calpain and NMDAR, inhibition of glutamate-stimulated Bax expression, and also effectively docked to NMDAR and GSK-3*β* [77].

In Alzheimer's disease (AD), vitexin and its analogs have been demonstrably shown to possess novel medicinal benefits. Cholinesterase (ChE) breakdown acetylcholine (Ach) in the central nervous system, and the reduced levels of ACh in the brain are implicated in AD. Substantial data have shown that vitexin-4′-O-glucoside, vitexin, isovitexin, and isovitexin-4′-O-glucoside (isosaponarin) suppressed the catalytic role of acetylcholinesterase (AChE) and butyrylcholinesterase (BChE) [42, 78–80]. Further docking analysis explained that vitexin interacts with AChE through hydrogen bonds [78]. In addition, vitexin favorably attenuated the function of *β*-site amyloid precursor (APP) cleaving enzyme 1 (neuropathological marker of AD) and generation of ROS [42, 78, 79]. Malar et al. [77] illustrated that vitexin downregulates glutamate transporters and protect neuro-2a cells from glutamate toxicity involved in AD. Furthermore, vitexin showed protection against memory impairment by enhancing memory retrieval and also reversed the shorter step-through latencies in AD [81]. More so, the anticonvulsant effect of vitexin was noticed in its possible ability to modulate minimal clonic seizures in pentylenetetrazol-induced rats and generalized tonic clonic seizures by delaying seizure appearance time [81, 82]. It is clear that there is some scientific proof to support the antineuropathy properties of vitexin and isovitexin analogs.

#### 3.2.6. Hypothalamus-Pituitary-Gonadal Axis and Dopamine D-2 Receptors

Recently, sexual dysfunction and fertility impairment cases have attracted attention as one of the major complications of diabetes. The hypothalamus-pituitary-gonadal axis have been suggested as the major pathway linking sexual dysfunction and fertility impairment to diabetes in male animals and humans [83]. The work by Li et al. [84] revealed that vitexin rectifies sexual dysfunction and fertility impairments in male diabetic mice possibly via modulating the hypothalamus-pituitary-gonadal axis. According to this report, vitexin significantly enhanced the sexual behavior, fertility levels, testicular pathological structure damage, and reproductive organ weight and restored sperm quality compared with the diabetic control group. Further downstream analysis indicated that vitexin increased secretion of serum testosterone, follicular-stimulating hormone, and luteinizing hormone contents, whereas the gonadotropin-releasing hormone level was decreased. Also, in a study involving the treatment of premenstrual symptoms, vitexin and isovitexin were screened against dopamine D-2 receptors that regulate prolactin synthesis, and it was observed that vitexin and isovitexin inhibited dopamine D-2 receptor at higher concentration [85]. These experimental results provide some indications as to the impact of these flavonoids on endocrine disorders which result in persistent hyperglycemia and as such, further investigations are on its use as a possible drug agent in sexual dysfunction and fertility impairment management. The multifaceted medicinal effects of these flavonoids deserve more investigative studies to further understand its pharmacological role in sexual dysfunction and fertility impairment.

#### 3.2.7. AGEs, Kidney Injury Molecule-1, and Neutrophil Gelatinase-Associated Lipocalin

Diabetic nephropathy (DN) is a leading cause of end-stage renal failure and contributes to most chronic complications of DM. Blocking the formation of advanced glycation endproducts (AGE) remains one of the therapeutic metabolic strategies in preventing DN and vascular diseases. Fortunately, it has been reported that isovitexin showed excellent inhibitory activities towards the formation of AGEs [43]. Additionally, other published studies have shown that vitexin and isovitexin exhibit their antidiabetic effects by inhibiting the formation of AGEs in vitro [42, 86].

Furthermore, the protective effect of vitexin on LPS-induced acute kidney injury and renal failure was recently observed and reported by Wang and colleagues [87]. The mechanistic studies illustrated that vitexin strongly reduced contents of serum creatinine and blood urea nitrogen, kidney injury molecule-1, and neutrophil gelatinase-associated lipocalin. Also, it upregulated AMPK/FOXO3a signaling via stimulating p-AMPK to AMPK ratio and protein content of FOXO3a in the nucleus while deactivating FOXO3a expression in cytoplasm. In DN, changes in MMPs expression add to extracellular matrix accumulation and glomerular hypertrophy which eventually cause proteinuria and renal insufficiency [88]. Testing for MMPs inhibitors, isovitexin and vitexin displayed the most potent inhibitory activity against MMP-1 expression at 100 lg/ml [89]. Moreover, vitexin and isovitexin were evaluated on the subtypes of MMPs (MMP-2, MMP-8, and MMP-9) using experimental and computational approaches. The two compounds showed pronounced inhibition activity towards the MMPs tested, and docking analysis revealed that vitexin and isovitexin bind to the active sites of the three tested MMPs [33]. In the I/R injury model, vitexin also protected BBB integrity, enhanced tight junction proteins synthesis, as well as inhibited MMP involved in regulating junction adhesion proteins [90]. Data portray the significance of vitexin and isovitexin as drug candidates for management of kidney damage.

#### 3.2.8. IL-1*β*, NF-*κ*B, and JUN N-Terminal Kinase (JNK) Pathways as Inflammation Targets

Diabetes mellitus triggers inflammation and oxidative stress which causes injury to body organs, resulting in numerous diabetic complications [91]. Existing evidence propose a possible role for inflammation in the pathogenesis of diabetes mellitus. The suggested mechanisms linking inflammation to diabetes comprise activation of IL-1*β*, NF-*κ*B, and JUN N-terminal kinase (JNK) pathways and recruitment of immune cells resulting in insulin resistance and dysfunction insulin secretion [92]. Therefore, targeting and blocking any of these inflammatory mechanisms represents a novel therapeutic approach for DM. Immune response modulatory property of vitexin, vitexin-2-O-rhamnoside, and vitexin-2-O-xyloside has been illustrated [93]. More so, topical anti-inflammatory activity of vitexin and isovitexin was previously stated [94]. The anti-inflammatory potential of vitexin measured in albumen denaturation inhibition assay was 54.2% compared to aspirin (55.6%), while vitexin inhibited proteinase by 57.8%, and the anti-inflammatory effect was obvious in vivo [95]. In animal studies, vitexin mitigated LPS-induced recruitment of neutrophils, maintained cytokine content, and activated nucleotide-binding domain and leucine-rich repeat PYD-containing protein 3 (NLRP3) inflammasome [75]. Better still, using a bioassay, the flavonoid 6‴-(3-hydroxy-3-methylglutaroyl)-2″-O-*β*-D-galactopyranosyl vitexin inhibited complement activation on the classic pathway in vitro [96].

The NF-kB molecule is a transcription factor that controls the transcription of DNA for the perpetuation of the inflammatory immune response and acts as a switch for turning inflammation on and off in the body [92]. The NF-kB proteins are localized in the cytoplasm. Inhibitors interact with these proteins and hijack them from directing DNA to express inflammatory proteins [92]. As an anti-inflammatory agent, administration of vitexin drastically attenuates the I/R-induced increased myocardia NF-*κ*Bp65 proteins content compared with control mice [86, 97].

It was previously reported that mast cells are activated by physical contact with activated T cells [98]. Following activation, mast cells discharge a group of inflammation mediators, including IL-1, IL-6, IL-8, IL-10, and TNF-*α* [98], and excessive induction is implicated in numerous metabolic disorders. Targeting inflammatory factors using IL-1 antagonists in patients with T2D, during clinical trials, showed promising results and support the role of inflammation in diabetes [92]. In both Jurkat T lymphocytes and RBL-2H3 mast cells, it was shown that vitexin inhibited the calcium release-activated calcium channel currents (ICRAC) and may inhibit mast cell degranulation and T cell recruitment [99]. Independently, vitexin and 2″-O-*α*-L-rhamnopyranosyl vitexin markedly decreased the migration of leukocyte in vivo and reduced particularly the peritoneal lavage neutrophils expression [5, 100]. In both in vivo and in vitro models, vitexin and 2″-O-*α*-L-rhamnopyranosyl vitexin treatment suppressed the secretion of activator-inflammatory cytokines TNF, IL-6, and IL-1*β* proteins' content compared with controls [5, 55, 97, 100–103]. Yang et al. [104] suggested that the vitexin inhibitory effect on IL‐1*β*‐induced inflammatory responses and may be attributed partly to the inhibition of the HIF‐1*α* pathway. In the inflammation model, vitexin exhibited pronounced activity in blocking the expression of TNF-*α* and the subsequent neutrophil activation [74, 105, 106]. Hashiguchi et al., in 2017 [107], reported that treatment of macrophage cells with vitexin alone did not change the expression level of IL-6; however, a combination of mung bean coat extracts with additional vitexin suppressed IL-6 expression in cells. Interleukin-10 (IL-10) is a potent anti-inflammatory cytokine with properties that suppress the overprovocation of host immune response, averting injury to host tissues and normalization of tissue homeostasis [108]. IL-10 dysregulation, especially during potent immune response, is related to increased immunopathology as well as high risk for acquisition of autoimmune syndromes [108]. Nevertheless, vitexin possessed the property to directly stimulate the synthesis of IL-10 to attenuate the mounted inflammation process either in inflammatory models or in vitro [5, 101].

The major pathways such as extracellular-regulated protein kinases 1/2 (ERK1/2), P38, and c-Jun N-terminal kinase (JNK) signals in the MAPK signaling pathway are associated with the pathogenesis of hyperinsulinemia in DM. Dysregulation of ERK1/2, P38, and JNK became a logic treatment step in chronic inflammation [109]. Vitexin has the property to reduce the expression of p-p38, p-ERK1/2, and p-JNK in macrophages [5]. Vitexin decreased progression of neutrophils to inflammation by targeted downmodulating proinflammatory regulators achieved through suppression of p38, ERK1/2, and JNK classical pathways [110].

The medicinal target, 5-lipoxygenase, catalyzes the biosynthesis of inflammatory mediators (leukotrienes), in chronic inflammatory diseases [111]. In an invitro study, 5-lipoxygenase enzyme was shown to be moderately inhibited by vitexin with IC_50_ = 5.1 *μ*M, referenced to indomethacin (IC_50_ = 0.98 M) [112]. Vitexin exerted a powerful reduction of adenosine deaminase and myeloperox expression as they relate to the release of cytokines (TNF-*α* and IL-17) in mice [113]. Myeloperoxidase (MPO) is noticed to be a mediator of inflammation and tissue injury, resulting in autoimmune syndromes. Multiple studies have observed the reduction of myeloperoxidase function using saponarin, vitexin, and isovitexin in vitro [106, 114].

Cyclooxygenases (COX-1 and COX-2) and prostaglandin E2 (PGE2) are inflammatory mediators. The COX enzymes catalyse the breakdown of arachidonic acid into prostaglandins, and attenuation of COX with NSAIDs leads to declined PGE2 release and decreased inflammation. The anti-inflammatory effect of vitexin via COX-1, COX-2, and PGE2 modulation has been studied. In macrophages, vitexin and isovitexin suppressed COX-1 and COX-2 mRNAs expression [103, 115–117]. A recent study illustrated that vitexin displayed moderate inhibition more towards COX-2 compared to COX-1 enzyme, and the flavonoids interacted with hydrogen bonding more at the cyclooxygenase catalytic sites just like celecoxib, the known inhibitor [118]. An in silico study disclosed that vitexin had the highest fitness score against the COX-2 (63.49) and for enzyme COX-1 (60.43) and showed tactical hydrogen bonding against COX-1 and COX-2 [119]. Furthermore, vitexin and isovitexin have the property to suppress PGE_2_ in macrophages [5, 117]. Available evidence affirms the role of vitexin and isovitexin in inflammation in the pathogenesis of diabetes mellitus. Thus, they could be considered in clinical trials research as a prophylaxis drug agent in diabetes mellitus.

#### 3.2.9. Reactive Species

The wide-ranging involvement of reactive species or free radicals and accompanied declined antioxidant capacity in most diabetic complications is well established. Reactive species are derivatives of normal cell function, and overproduction accelerates hyperglycemia-mediated oxidative tissue damage implicated in human diseases including metabolic and cardiovascular disorders [120]. Data from earlier studies showed that antioxidants diminish the rate of developing diabetic-related complication and recover insulin sensitivity [120]. The mechanistic approach to antioxidant activity by vitexin and its derivatives appears in three ways. One is by limiting the expression and/or the activities of enzymes that directly catalyse the production of oxidants, second, upregulate the expression and/or activity of enzymes involved in the generation of cellular antioxidants, and finally, acts as ROS scavengers themselves.

During excessive ROS discharge, the expression levels of lactate dehydrogenase (LDH) and creatine kinase (CK) are upregulated. Treatment with vitexin alleviates oxidative stress via decreasing the release of ROS and downregulates the synthesis of LDH and CK enzymes [73, 121–124]. In the course of lipid peroxidation, malondialdehyde (MDA) production increases due to increased oxidative stress; nonetheless, vitexin and vitexin-4″-O-glucoside have the properties to suppress lipid peroxidation and significantly reverse the enhanced production of MDA [73, 121–123, 125]. In neuro-2a cells, elevated production of protein carbonyl content (PCC) and MDA and endoplasmic reticulum stress gene (Gadd153) induce ROS-mediated toxicity. However, pretreatment with vitexin restored PCC and MDA levels, significantly downregulated Gadd153 (0.93 fold) leading to the reduction of free radicals, suppressed ROS-mediated lipid peroxidation and protein oxidation, and protects membrane potential [70]. Several studies have confirmed the lipid peroxidation properties of vitexin, vitexin-4″-O-glucoside, and isovitexin in vitro [126–129]. NO synthase (NOS) catalyse the breakdown of l-arginine to form nitric oxide (NO) which reacts with reactive superoxidefree radical to form toxic peroxynitrite which has tissue damaging properties. Culturing lipopolysaccharide- (LPS-) triggered macrophages in the presence of vitexin attenuates stimulated NO release and synthesis of nitric oxide synthase (iNOS) in the macrophages [130]. Intracellular NO and ONOO^−^ production are modulated by eNOS and iNOS activities, and vitexin demonstrates its antioxidant effect via powerful reduction of LDH and iNOS function as well as escalating eNOS phosphorylation and activity in cells [90]. During in silico docking experiment to NOS and xanthine oxidase (superoxide-generating enzyme), isovitexin presented great potential in the docking and binding to the enzymes targeted; whereas, vitexin docked and bound with only NOS [131]. Furthermore, myeloperoxidase-derived oxidants serve as a vital contributor to tissue damage, and reports showed the reduction of myeloperoxidase and horseradish peroxidase catalyzed oxidation by saponarin, vitexin, and isovitexin [106, 114]. Vitexin and isovitexin have potent antioxidant activities with effective inhibition of advanced glycation end products [86]. Also, xanthine oxidase, 15-lipoxygenase, and nitric oxide synthase enzymes induce oxidative modification by generating ROS during their catalytic activities, leading to uncontrolled tissue damage. Isovitexin has been reported to inhibit these enzymes activities [132–134].

In the body, protection against oxidative stress is achieved by antioxidants generally generated enzymatically including catalase (CAT), SOD, glutathione peroxidase (GPX), glutathione reductase (GR), and glutathione oxidase (GPX)) or via nonenzymatic sources glutathione (GSH), vitamin C, vitamin E, selenium, Zn, carotene, and beta-carotene. However, under oxidative stressed conditions, these enzymes are significantly repressed, exposing tissues and organs to injury. One of the antioxidant mechanisms of vitexin is to increase the activities of these enzymes, thereby discharging antioxidant that efficiently quench reactive species and limit ROS content in the system. In in vivo, it was observed that pretreatment with vitexin and vitexin-4″-O-glucoside significantly promoted CAT and SOD activities [73, 122, 125, 135–137]. Also, upregulation of FOXO3a stimulates the production of antioxidant enzymes, and vitexin is known to activate the expression of p-FOXO3a in the rat model [124], thus demonstrating a medicinal effect against free radicals-induced body injury. The protein NADPH acts as an indicator of oxidative status and has a positive effect on the cellular antioxidant potentials; meanwhile, vitexin has the property to increase NADPH protein expression levels and exerts lipid peroxidation impact in I/R injury models [121]. Vitexin nullifies oxidative stress via decreasing the discharge of ROS whilst promoting the activity of glutathione peroxidase [73]. Nuclear factor erythroid-2-related factor 2 (Nrf2) is believed to control expression of antioxidant enzymes, e.g., heme oxygenase- (HO-)1 which protects organs against oxidative damage [138]. In in vivo and in vitro, vitexin and isovitexin enhanced the expression of Nrf2 and HO-1 activity and attenuated oxidative stress injury [75, 76]. Work performed by Malar et al. illustrated that vitexin mitigated induce ROS-mediated toxicity by significantly activating endoplasmic reticulum stress gene, Grp78 (0.84 fold) [70].

In the third antioxidant mechanism, vitexin and its derivatives exhibit their antioxidant capacities by rapidly quenching the attacking ROS or NOS and thereby alleviating their destructive power. Several studies on antioxidant potentials of these glycosides in different antiradical assays have been reported. Growing evidence support that vitexin scavenge NO in vivo or in vitro, thereby eliminating its destructive pathways [5, 74, 102, 106, 139]. In the DPPH method, absorbance of DPPH radical decreases as the radical is scavenged by antioxidants through donation of hydrogen to form the stable DPPH-H. The more rapid the decrease in absorbance, the more potent the antioxidant activity of the compound in terms of hydrogen donating ability [140]. Antioxidant activity of vitexin and its analogs were investigated using the DPPH assay and isovitexin-6″-O-*α*-L-glucoside, vitexin, vitexin-2″-O-rhamnoside, isovitexin, 2″-O-rhamnosyl vitexin, vitexin-2-O-rhamnoside, vitexin 2″-O-xyloside, isovitexin-7-O-glucoside, isovitexin 6″-O-E-p-coumarate, and vitexin 2″-O-glucoside exhibited significant DPPH• scavenging activities [128, 132, 141–147]. Vitexin and isovitexin DPPH radical scavenging activity is suggested to be attributed to the phenolic hydroxyl group in the 4′ position on the B-ring [33, 38, 143, 146, 148–157]. Moreover, isovitexin-6″-O-*α*-L-glucoside, vitexin, vitexin-2″-O-rhamnoside, and isovitexin demonstrated moderate to high affinity to generated free ABTS•+ in the ABTS assay [44, 141–145, 156–162]. In superoxide radical-scavenging assay, isovitexin and vitexin showed a protective effect against the oxidative-induced reactions by scavenging generated free superoxide anion radicals [37, 133, 147, 150, 156, 163, 164]. Vitexin andvitexin-2″-O-*p*-trans-coumarate have anti-ROS protective influence against H_2_O_2_-mediated oxidative injury, sensitive in neutralizingfree oxygen radical, and also offers protection to the antioxidant enzymes [133, 161, 165, 166]. The 7-OH and 4′-OH groups were suggested to be important in the activity [167]. Furthermore, in *β*-carotene bleaching assay, isovitexin and vitexin showed moderate to strong antioxidant effects in inhibition of *β*-carotene bleaching [146, 151, 168]. Isovitexin and vitexin have also demonstrated ferric reducing capacities [128, 133, 151, 169] and Fe II chelating abilities [160, 161]. Vitexin 2″-O-*β*-D-xylosyl vitexin and 2″-O-galloyl vitexin exhibited the strongest antioxidant activity in ORAC assay [170, 171].

Nevertheless, other studies claimed that vitexin and isovitexin did show weak to no significantfree DPPH radical scavenging abilities [33, 84, 127, 141, 142, 153, 158, 159, 169, 172–181]. Also, poor antioxidant activity of vitexin in scavenging ABTS•+ have been observed [153, 161]. In addition, vitexin and isovitexin were weak scavengers of ClO− in copper-phenanthroline assay [161]. Vitexin-2-O-rhamnoside showed low power in scavenging freely generated radicals in tyrosine nitration inhibition assay [142]. Whereas, studies by von Gadow et al. [182] and Leong et al. [152] observed isovitexin and vitexin to be weak antioxidants in the *β*-carotene bleaching test and further explained that the observed is due to isovitexin and vitexin not having the 3′,4′-dihydroxy configuration or m-5,7-dihydroxylation of the A-ring, which provides good electron delocalization and stabilization of phenoxy radicals and the compounds with only one hydroxyl group on the B-ring.

The chemical structures of vitexin and its analogs greatly influence the antiradical properties of these compounds. It is proposed that the antioxidant capacity of these compounds depend on the extent of hydroxylation, position of glycosylation, and the kind of glycosylation [94, 156, 183, 184]. Additionally, glycosylation of the A-ring and/or loss of the 3-OH distinctly affects the antioxidant activity of vitexin, while mono OH substitution at 4′ of vitexin has stronger antioxidant activity [164, 171, 185, 186]. Moreover, glycosylation at C-6 of the A-ring makes greater uniform distribution of spin density as well as enhances stability of free radicals resulting in superior antioxidant capability [187]. In contrast, Lespade and Bercion [188] reported that glycosylation causes charge changes on the oxygen atoms of the hydroxyl groups and generally leads to a decrease in the antioxidant ability of a compound; thus, glucose substitution at position 7 leads to a decrease in negative charge on the oxygen atom at position 3, which is not exciting for potent antioxidants even if it leads to a slightly better antioxidant potency. The most potent antioxidants have a large negative charge on the oxygen atom which gives out its hydrogen [188]. Also, Ma et al.[189] proposed that introducing an acyl group into isovitexin significantly improved its lipophilicity but reduced its antiradical activity, particularly, acylation at the isovitexin primary hydroxyl group of glucose moiety. This lengthened exposition on the antioxidant properties of vitexin and isovitexin clearly affirms their role as antioxidants. Thus, they could be considered for further research as alternatives to some synthetic antioxidants reported to have many adverse effects.

#### 3.2.10. Cell Signaling Molecules Involved in Apoptosis, Autophagy, Mitochondrial, and Platelet Aggregation

In diabetic wounds, cell proliferation, differentiation, and angiogenesis are profoundly disturbed which impairs wound healing [190]. The wound healing property of vitexin was recently published [191]. The wound healing effect of chitosan-based gel formulation containing vitexin was assessed, and the gel potently accelerated wound healing both in vivo and in vitro. Histological examinations confirmed that vitexin formulation triggered skin regeneration and that the wound healing assay showed vitexin-containing gel to strongly activate cell proliferation.

Diabetes occurs more often in individuals with chronic obstructive pulmonary disease (COPD) than in the general population; for that matter, diabetes has been proposed as the risk factor of COPD [192]. Managing COPD is recognized as a logic step in reducing some of the morbidities of diabetes. Flavone C-glycosides, vitexin, and isovitexin exhibited potent inhibitory activities against IL-8 production and matrix metalloproteinase-1 (MMP-1) expression, an inflammatory marker for COPD [89]. In both assays at 100 *μ*g/ml, the two compounds demonstrated their potential therapeutic role in the treatment of COPD by preventing the pathological disruption of the lung extracellular matrix observed in this disease.

DM individuals pose approximately four times higher risk for stroke, and cardiometabolic risk factors such as hypertension, obesity, and dyslipidemia [193]. A study reported that vitexin could suppress the autophagy dysfunction by attenuating middle cerebral artery occlusion-induced cerebral ischemic stroke via the mechanistic target of rapamycin, the mTOR/Ulk1 pathway, thus alleviating oxidative injury and reducing the production of proinflammatory mediators [101].

Diabetes triggers inflammation and oxidative stress which cause injury to body organs, resulting in numerous diabetic complications [91]. The pharmaceutical effect of vitexin on DM-induced cardiovascular diseases is accomplished through protein kinases, the key cellular modulators of glucose homeostasis and AMPK [137], PI3K/Akt/mTOR [123], and MAPKs [194] signaling pathways. AMP-activated protein kinase (AMPK) is a glucose-sensing enzyme that is triggered when cellular glucose levels are low, and it signals to stimulate glucose metabolism in muscles, fatty acid oxidation in adipose tissues, and decreases hepatic energy accumulation [195]. AMPK activation prompts insulin-sensitizing effects, making it an ideal therapeutic target for t2ds. Multiple evidences suggest that AMPK is dysregulated in DM; however, vitexin directly induced AMPK activity at low *μ*M concentrations, which suppressed intracellular and plasma cholesterol contents in HepG2 cells and mice [135]. In the AMPK signaling pathway, vitexin prevented cell injury by prompting autophagy, enhanced cell viability, and alleviated apoptosis in the cells [137]. At the cellular level, vitexin enhanced autophagy via stimulating the synthesis of p-AMPK while reducing p-mTOR expression, and the protective effects of vitexin were blocked by applying autophagy inhibitor to cells. In addition, vitexin suppressed the expression of proapoptotic markers such as Bax, cleaved caspase-3, and cleaved caspase9; whereas, Bcl-2 expression was enhanced. Furthermore, vitexin also elicited the anti-inflammatory effect through inhibiting the overexpression of VCAM-1, ICAM-1, TNF-*α*, E-selectin, IL-1, and IL-6, and finally, vitexin alleviates oxidative stress by promoting cellular antioxidant activities [137].

The apoptotic protective property of vitexin also works by using the MAPK signaling pathway in vitro and in vivo. Lyu et al. [73] indicated that vitexin treatment activates expression of the hypoxia inducible factor-1*α* subunit (HIF-1*α*) and vascular endothelial growth factor (VEGF) proteins and blocked expression of phosphorylated-p38 MAP kinase (p38) protein in rat. Better still, vitexin suppressed Bax, cleaved caspase-3, NF-*κ*B, and TNF-*α* production and nullifies oxidative stress via stimulating the activities of antioxidant enzymes [73, 77, 122]. The cardioprotective effect of vitexin is reported to be achieved by inhibiting the initial activation of ERK and p38 MAPK pathways through suppressing the activation of NFATc1, c‐Fos, and phospho-c-Jun and at the same time enhancing the expression of phospho-ERK [122, 196]. Bhardwaj et al. [194] suggested that during heat stress, vitexin binds to HSF-1, enhances cell viability, upregulates Hsp90 expression, and thereby activates ER-stress induced autophagy, consequently modulating MAPKs expression and leading to vacuole accumulation and autophagic flux in cells. In HCT-116 cells, exposure to vitexin recruits HSF-1 target proteins downstream and inhibits heat shock proteins [197]. Further experiments identified HSF-1 as a possible molecular target of vitexin. It binds to the DNA-binding domain in HSF-1 protein, which suppresses oligomerization and activation of HSF-1 [197].

In preventing cardiovascular injury, vitexin mitigated apoptosis and autophagy in myocardium cells by the use of PI3K/Akt/mTOR signaling pathway [123]. In H9c2 cells, vitexin inhibited autophagy by suppressing apoptosis and reducing Beclin1 and LC3I/II expression, while p62 expression was upregulated. Vitexin suppressed apoptosis by upregulating Bcl-2, whereas the synthesis of cleaved caspase-3, Epac1, Rap1, and Bax was suppressed [121, 123]. In addition, vitexin also activated the cellular antioxidant pathway [121, 123, 124]. Again, vitexin inhibited the expression of inflammatory cytokines, attenuated increased levels of IL-6, TNF-*α*, and IL-1*β*, and downregulated NF-*κ*Bp65 [97, 124].

Vitexin protected mitochondrial dysfunction by promoting mitochondrial activity, its membrane activity, and the ATP level, while it ultimately mitigated mitochondrial injury and cardiomyocyte apoptosis through enhancing the expression of MFN2, blocked the induction of Drp1, and significantly mitigated ROS content [198]. Moreover, in vitro, treating genotoxic-induced larvae with vitexin prompted its antigenotoxicity effects and decreased the occurrence of mutant spots, compared to control [199].

Vitexin protection of RGC-5cells from oxidative stress injury and death has been illustrated. Treatment of vitexin to RGC-5 cells induced with either l-buthionine-(S,R)-sulfoximine and glutamate or H_2_O_2_ prevented cell death by mitigating ROS oxidative effects, but the activity was low [200]. Hence, the therapeutic use of vitexin in retinal degenerative disease is repurposing. Lei and Yang reported the cure of the atherosclerosis rat model by feeding with vitexin for 30 days [201]. Further experimental analysis showed that feeding with vitexin or pravastatin or incombination attenuated increase in serum IL-1b, IL-6, TNF-*α*, total cholesterol, atherogenic index, low-density lipoprotein, ICAM-1, MCP-1, VCAM-1, aortic nitro tyrosine, and liver HMG-CoA-reductase activity. Also, vitexin restored endothelial function and decreased in serum high-density lipoprotein, %HTR (HDL/TC ratio), PON1, homocysteine thiolactonase, aortic SOD, GPx, and CAT.

Platelet aggregation is the drug target in the cure of most vascular diseases such as acute coronary syndrome, stroke, ischemic attacks, peripheral artery occlusive, and coronary disease in DM [202]. The management of these diseases usually involve the use of platelet aggregation inhibitors that attenuate activation and/or aggregation of thrombocyte [202]. Several studies have reported the platelet aggregation inhibitory properties of vitexin and its analogs. The antiplatelet effects of vitexin was tested, and vitexin showed pronounced biological activity by suppression of platelet aggregation mediated by collagen or ADP [203]. In rat plasma and in transgenic zebrafish, vitexin and 2″-O-rhamnosyl vitexin markedly inhibited platelet aggregation; furthermore, 2″-O-rhamnosyl vitexin inhibited thrombus production induced by FeCl3, and the compounds antiplatelet activities were considered to be acting through the adenosine diphosphate pathway [204]. Additionally, isovitexin 6″-O-glucoside and isovitexin produced moderate antiplatelet activity and suppressed arachidonic acid, collagen, thrombin, and platelet-activating factor in rabbit platelets [205].

### 3.3. Safety and Toxicity Profile of Vitexin and Isovitexin

The elucidation of the safety and toxicity profile of small molecules is an integral stage of drug discovery and development. Substantial and growing data now exist concerning the toxicity and safety profiles of vitexin and its derivatives. The toxicity of vitexin and its derivatives have been observed by (1) studies in vitro using cells or cell lines and (2) in vivo experimentation in animal models. The cytotoxicity of vitexin and isovitexin has been assessed in human leukemia CCRF-CEM cells [206], macrophages cells (RAW264.7) [134, 207], T lymphocytes and RBL-2H3-mast cells [99], N9 microglial cells [74], neutrophils [106], breast carcinoma cell lines, either T47D or MCF-7 [203], MCF-7, A549, HepG2, and HT-29 cells [208], HepG2 cells [209], J774 macrophages cells [139], and human keratinocyte (HaCaT) cells [144], and they only exerted negligible to no cytotoxic effects. In addition, vitexin-2″-O-rhamnoside and vitexin-4″-O-glucoside caused no cytotoxic effect on human adipose-derived stem cells, A2780 and MCF-7cells [210, 211]. Studies in neuro-2a cells revealed that A-*β*25-35 significantly affected the cell viability by inducing ROS-mediated toxicity and apoptosis. However, pretreatment of neuro-2a cells with vitexin (50 *μ*M) significantly restored the cell viability up to 92.86 ± 5.57% [70]. On the contrary, 100 and 500 *μ*M isovitexin concentrations were found to be toxic to Caco-2cells after 4 h of incubation [212]. The acute cytotoxicity of vitexin and isovitexin was evaluated in both normoglycemic mice and induced diabetic rat, and the results show that at 2 g/kg wt, neither the normoglycemic mice nor diabetic rat showed any overt signs of toxicity after 24 h and 14 days of observation, and the weight of the rodents was not significantly different with no mortality recorded throughout the 14-day monitoring [19, 20].

For the genetic toxicity test, the results showed that vitexin insignificantly induced few mutant spots and exhibited no genotoxic activity in vitro [199]. The antimutagenic potentials of flavones, isovitexin, and vitexin were compared in the *Salmonella typhimurium* mutagenicity assay using tester strains TA100 and TA98 and mutagens such as aflatoxin B1 (AFB1) and 2-acetamido-fluorene (2-AAF) alongside with metabolic stimulation. From data, vitexin showed a protective effect at 0.4 mM but, however, lost this protection at 0.08 and 0.8 mM against 2-AAF. On the other hand, isovitexin in a dose-dependent manner enriched 2-AAF mutagenicity at concentrations greater than 0.4 mM. Both flavones have protective activities on AFB1 at higher concentrations ≥0.4 mM [213]. Structural features that affect antimutagenic capacities of natural flavonoids include physiochemical parameters such as hydrophilicity or lipophilicity which is dependent on the degree of hydroxylation as well as O-methylation, glycosylation on rings A and B, C4-keto moiety, and the double bond at C2–C3 [213]. Furthermore, the in vivo genotoxic test, SMART, in *Drosophila melanogaster* wings demonstrated that vitexin has no genotoxic activity [199]. The metabolite vitexin was investigated to establish genotoxic profiles in viable human lymphocyte cells in vitro for 48 h, and the results show that the planar flavones vitexin enhances the sister chromatid exchange frequency by a factor of around 4-5 at a concentration of 100 *μ*g/ml and induces micronuclei and a small increase in polyploid cells [214].

## 4. General Discussion

**Figure 3:**
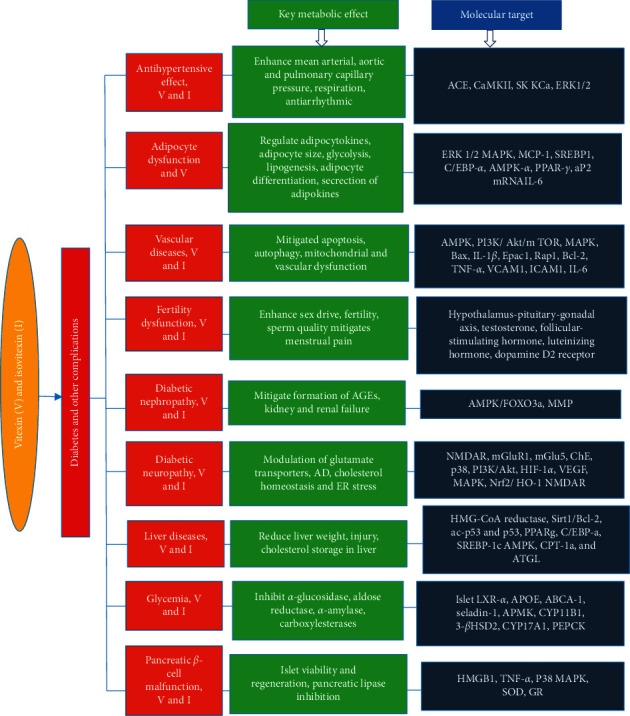
Schematic diagram of metabolic effects and molecular targets of vitexin and isovitexin in diabetes mellitus and its complication. V, vitexin; I, isovitexin.

Despite the fact that isovitexin and vitexin both possess a similar molecular weight of 432.381 g/mol, their analogs vary considerably in terms of their huge masses. This characteristic could contribute to the differences observed among these compounds. The current review sums up the efficacies of vitexin and its analogs for therapeutic application in diabetes and its complications considering multiple drug targets that may be involved. Numerous reports reviewed outlined the molecular mechanisms of vitexin and isovitexin analogs in diabetic-related complications. There appears to be clear evidence that vitexin and its analogs possess many pharmacological benefits in attenuation of diabetic complications including adipose tissue dysfunction, sexual and fertility impairment, pancreatic *β*-cell malfunction, hyperglycemia, diabetic neuropathy, liver disorders, diabetic nephropathy, vascular disease, platelet aggregation, and hypertension. Data points to the fact that the antidiabetic mechanism of vitexin and its analogs acts mainly by alleviation of cell apoptosis, targeting point in hypothalamus-gonadal axis and other organs affected by persistent hyperglycemia. Additionally, oxidative stress due to their inflammation is also controlled (Figure 3). Again, substantial evidence has proven the safety and low toxicity profiles of these compounds in vivo and in vitro. Further studies are required to outline additional scientific evidence of vitexin and its analogs for clinical trials in the treatment of diabetes and associated sicknesses. For now, vitexin research on retinopathy is limited, and hence, more studies in that regard would add to knowledge. There are no clinical trial studies reported on only vitexin and isovitexin in diabetes mellitus. The only available work was the inclusion of vitexin in a dietary supplement named Ritmonutra® used to prevent benign supraventricular and ventricular arrhythmias in people who are free of a specific heart disease (http://www.ClinicalTrails.gov).

## 5. Concluding Remarks

It is clear that vitexin and its analogs possess several pharmacological relevances in attenuation of diabetes mellitus and its complications. In addition, the antidiabetic mechanism of vitexin and its analogs is through alleviation of cell apoptosis and oxidative stress due to their anti-inflammation and antioxidant properties. Also, ample evidence has showed the safety of these compounds in vivo and in vitro. Available published reports support the multifaceted medicinal role of vitexin and its analogs in managing diabetes mellitus and its complications.

## Data Availability

The datasets used to support the findings of this study are included within the article.
